# The role of matrix metalloproteinase-9 as a prognostic biomarker in papillary thyroid cancer

**DOI:** 10.1186/s12885-018-5112-0

**Published:** 2018-12-03

**Authors:** Maryam Zarkesh, Azita Zadeh-Vakili, Mahdi Akbarzadeh, S. Ahmad Fanaei, Mehdi Hedayati, Fereidoun Azizi

**Affiliations:** 1 Cellular and Molecular Endocrine Research Center, Research Institute for Endocrine Sciences, Shahid Beheshti University of Medical Sciences, Po Box: 19395-4763, Tehran, Iran; 2 Cellular and Molecular Endocrine Research Center, Research Institute for Endocrine Sciences, Shahid Beheshti University of Medical Sciences, Po Box: 19395-4763, Tehran, Iran; 3 Cellular and Molecular Endocrine Research Center, Research Institute for Endocrine Sciences, Shahid Beheshti University of Medical Sciences, Tehran, Iran; 4 Association Professor of General Surgery, Erfan Hospital, Tehran, Iran; 5 Cellular and Molecular Endocrine Research Center, Research Institute for Endocrine Sciences, Shahid Beheshti University of Medical Sciences, Tehran, Iran; 6 Endocrine Research Center, Research Institute for Endocrine Sciences, Shahid Beheshti University of Medical Science, Tehran, Iran

**Keywords:** BRAF V600E, Matrix metalloproteinase-9, Papillary thyroid cancer

## Abstract

**Background:**

The aim of the present study was to investigate the association between matrix metalloproteinase-9 (MMP-9) expression with BRAF V600E mutation and clinicopathological features, in Iranian papillary thyroid cancer (PTC) patients.

**Methods:**

In total, 90 participants including 60 PTC patients (15 males and 45 females) and 30 individuals with benign multinodular goiter (MNG) (5 males and 25 females) which were confirmed by surgical pathology, were investigated. MMP-9 was evaluated at both mRNA and protein levels, using SYBR-Green Real-Time PCR and enzyme-linked immune sorbent assay (ELISA), respectively. BRAF V600E mutation was detected by sequencing.

**Results:**

Mean age of PTC and MNG patients was 37.6 ± 12.6 and 48.1 ± 13.3 years, respectively (*P* = 0.001). BRAF V600E mutation was found in 24 of the 60 (40%) PTC cases, with mean tumor size of 1.59 ± 1.20 cm. MMP-9 mRNA levels were elevated in tumoral compared to the adjacent non-tumoral tissues (*P* = 0.039); moreover, this rise was also observed in PTC patients compared to MNG patients (*P* = 0.001). The mRNA levels of MMP-9 increased in patients aged≥45 years (*P* = 0.015), those with lymphovascular invasion (*P* = 0.003), and higher tumor stages (III and IV) (*P* = 0.011). The protein level of MMP-9 increased in tumoral compared to adjacent non-tumoral tissues (*P* < 0.001); this increase was also found in PTC patients compared to MNG participants (*P* = 0.004). MMP-9 protein level was higher in patients aged≥45 years (*P* = 0.001), those with lymphovascular invasion (*P* = 0.036) and higher TNM stages (III and IV) (*P* = 0.001). Area under the ROC curve (AUC) was 0.70 (95%CI: 0.57–0.83, *P* = 0.003), with 91.4% sensitivity and 51.9% specificity at the cutoff value of 0.50.

**Conclusion:**

The mRNA and protein levels of MMP-9 had no association with BRAF V600E mutation in Iranian PTC patients. These levels were associated with age, TNM stages, and lymphovascular invasion, being defined as malignant factors. Thus, elevated levels of MMP-9 in PTC patients compared to MNG participants illustrated that it can be used as a potential biomarker to differentiate PTC patients from those with MNG.

## Introduction

Well-differentiated thyroid carcinomas are classified into papillary and follicular thyroid carcinoma (PTC and FTC), and are the most frequent endocrine malignancy, consisting over 70% of the cases [1]. The estimated incidence of thyroid cancer is > 3 fold higher in women [2].

Environmental, genetic and hormonal factors are the most important triggers in the etiology and increased prevalence of human cancers [1], with genetic mutations playing the most important role in tumorigenesis. Numerous oncogenes and rearrangements with different frequencies and properties have been implicated in the pathogenesis of PTCs, mostly involving the abnormal activation of RAS-RAF-MEK- mitogen-activated protein kinase (MAPK) pathway [3]. Of these, activating mutation in the B isoform of the Raf kinase gene, results in a valine to glutamic acid substitution at amino acid 600 (BRAF V600E) which occurs in PTC and PTC-derived anaplastic thyroid cancer (ATC) [4]. Its prevalence is highly variable, ranging from 29 to 83% among different reports [5]. In the previous study, its prevalence was reported as 40% in a sample of Tehran’s population [6]. In some studies, this mutation was correlated with advanced tumor or aggressive phenotypes; whereas, no such association was found in other studies [5, 7–10]. Moreover, it was found to be associated with larger tumor size and lymph node metastasis (LNM) in the Tehranian population [6].

Modifications in matrix metalloproteinases (MMPs) also play a main role in various human cancers, and can be involved in the differentiation, morphogenesis and tissue remodeling during angiogenesis, tumor invasion and metastasis [11]. One of the most significant MMPs is MMP-9, also known as 92-kDa gelatinase B type IV collagenase. Due to its complex regulation activity, it is involved in the pathogenesis of many diseases [12]. Some reports showed that the BRAF V600E can induce MMPs [13, 14]; a positive correlation has been reported between BRAF V600E and MMP-9 and MMP-2 expressions, both being correlated with tumor extrathyroid extension [15].

Evaluating the role of MMP-9 along with BRAF mutation can be useful in predicting the outcome of many cancers, especially PTC. Therefore, first, we tested the hypothesis that whether BRAF V600E mutation has an association with MMP-9 activity or not? We then studied the clinical relevance of MMP-9, in terms of invasion and metastasis among PTC patients also evaluating the predictive and prognostic role of MMP-9 levels.

## Material and methods

### Study population

In this case-control study, patients referred to Erfan and Atieh Hospitals (in Tehran, Iran), for near-total or total thyroidectomy, from November 2015 to August 2016, were initially enrolled. All patients with malignancies other than PTC were excluded based on postoperative pathological reports. Finally, 60 patients with PTC (45 females and 15 males) and 30 participants with benign multinodular goiter (MNG) (25 females and 5 males) were selected. Primary PTC tumor tissues, adjacent normal tissues from the same case (rather from another lobe) and normal tissues from MNG patients were collected during surgery by an expert surgeon. All confirmed thyroid samples were snap-frozen in liquid nitrogen and stored at − 80 °C. The demographic and pathological characteristics of participants were extracted from medical records. Tumor staging was determined using the 7th edition of the American Joint Committee on Cancer Tumor-Node-Metastasis (AJCC-TNM) staging system [16].

### Molecular assays

Genomic DNA and total RNA were extracted from collected fresh frozen thyroid tissues using the TRIzol reagent (Invitrogen U.S. Cat. No. 15596–026), after histological control and according to the manufacturer’s instructions. Genomic DNA was amplified by polymerase chain reaction (PCR) for exon 15 of the BRAF gene containing the site for the T1799A mutation in chromosome 15, which has been described previously [6].

Total RNA (1 μg) was reverse transcribed using cDNA synthetize kit (Thermo Fisher Scientific, USA) according to the manufacturer’s protocol and stored at − 20 °C for further use. To evaluate the MMP-9 expression, quantitative reverse transcriptase real-time PCR (qRT-PCR) was performed by Rotor-Gene 6000 instrument (Corbett Research, Sydney, Australia). The PCR was completed using the following thermal programs: initial denaturation (10 min at 95 °C) and then a three-step amplification program (15 s at 95 °C followed by 20 s at 60 °C and 40 s at 72 °C), with melting curve repeated 40 times. β-actin was used as the reference gene to normalize mRNA levels. All experiments were repeated twice. Sequences of the primers are shown in Table 1. PCR amplification was performed in 25 μL volumes using SYBR Green master mix (Thermo Fisher Scientific, USA).

**Table 1:** Primers information

Ref.	Genes	Primers	5′-3’	Tm	PCR product size (bp)
NM_004994.2	MMP-9	Forward	CTTTGAGTCCGGTGGACGAT	59	101
		Reverse	TCGCCAGTACTTCCCATCCT	60	
NM_001101.3	β-actin	Forward	GATCAAGATCATTGCTCCTCCT	57	108
		Reverse	TACTCCTGCTTGCTGATCCA	58	

### Enzyme-linked immune sorbent assay (ELISA)

Tissue MMP-9 protein levels were measured by a quantitative enzyme-linked immunosorbent assay (ELISA) method in three groups (PTC tumoral tissues, PTC adjacent non-tumoral tissues and MNG normal tissues). To extract total protein, collected tissues were incised and weighed (in total 100 mg tissue/ 1 mL buffer). A certain amount of PBS (pH 7.4, 100 mM) was added and homogenized thoroughly using a homogenizer (QIAGEN, Germany), and then centrifuged at 4000–6000 RPM for approximately 10 min; supernatants were collected and frozen at − 20 °C for later use. Research Human MMP-9 ELISA kit (Cat. No: ZB-0936-H9648) was obtained from ZellBio GmbH, Germany. Total protein level of MMP-9 was determined based on the sandwich ELISA method, by an ELISA microplate reader (Tecan Sunrise, Tecan, Austria), according to the manufacturer’s instructions. For normalization, the standard curve was drawn; the unit used for MMP-9 measurement was ng/μg. Intra-assay coefficients of variation (CV%) was < 10%.

#### Statistical analysis

Normal distribution of data was evaluated by the Kolmogorov-Smirnov (KS) test. The *x*^*2*^ test was used for group comparisons of categorical variables as frequency and percentage. For non-normal distributed data, the non-parametric Wilcoxon test was applied to compare the tumoral and adjacent non-tumoral tissues; the Mann-Whitney U test was used to evaluate the median of the two PTC and MNG groups. For normally distributed samples, the parametric Paired Samples t-test was used to compare the means of the tumoral tissues with the adjacent non-tumoral tissues; to assess the means of the PTC and MNG groups, the Independent Sample t-test was used. Normal distributed data were expressed as mean ± standard deviation (SD) and non-normally distributed data as median (inter quartile 25th, 75th). An age and sex adjusted logistic regression with hierarchical method was used to measure the effects of the independent variable (MMP-9 mRNA level and age) upon the dependent variables (TNM stage, lymphovascular invasion, and patients’ status) separately. For the final model, the goodness-of-fit model was examined using the Hosmer-Lemeshow test. The receiver operating characteristic (ROC) curve was drawn and area under the curve (AUC), sensitivity and specificity were calculated to assess the model precision and performance of diagnostic tests. PPV (positive predictive value) and NPV (negative predictive value) were calculated with sensitivity and specificity formula via MedCalc version 18.9.1. Statistical analyses were performed using SPSS 20.0 (Chicago, IL, USA) and graphs were plotted by MedCalc statistical software, with *P* value < 0.05 being considered as statistically significant.

Relative quantitation of MMP-9 mRNA levels was performed by the comparative Ct method, according to the following formula [17]:

$$ {\Delta  \mathrm{Ct}}_{\mathrm{Tumoral}\ \mathrm{tissues}}={\mathrm{Ct}}_{\left(\mathrm{MMP}-9\right)}-{\mathrm{Ct}}_{\left(\upbeta\ \mathrm{actin}\right)} $$

$$ {\Delta  \mathrm{Ct}}_{\mathrm{Adjacent}\ \mathrm{non}-\mathrm{tumoral}\ \mathrm{tissues}}={\mathrm{Ct}}_{\left(\mathrm{MMP}-9\right)}-{\mathrm{Ct}}_{\left(\upbeta\ \mathrm{actin}\right)} $$

$$ \Delta  \Delta  \mathrm{Ct}={\Delta  \mathrm{Ct}}_{\mathrm{Tumoral}\ \mathrm{tissues}}-{\Delta  \mathrm{Ct}}_{\mathrm{Adjacent}\ \mathrm{non}-\mathrm{tumoral}\ \mathrm{tissues}} $$

$$ \mathrm{Relative}\ \mathrm{expression}={2}^{-\Delta  \Delta  \mathrm{Ct}} $$

## Results

### Demographic and pathological characteristics

Demographic and pathological characteristics of the participants are presented in Table 2. The mean ages of PTC and MNG patients were 37.6 ± 12.6 and 48.1 ± 13.3 years, respectively which was significantly different (*P* = 0.001). The BRAF V600E mutation was found in 24 (40%) of the 60 PTC cases, with the mean tumor size being 1.59 ± 1.20 cm.

**Table 2:** Demographic and clinicopathological characteristics of participants

Parameters	PTC (%)	MNG (%)	Total (%)
Patients	60 (66.7)	30 (33.3)	90 (100)
Age			
< 45 years	43 (71.7)	10 (33.3)	53 (58.9)
≥45 years	15 (25.0)	17 (56.7)	32 (35.6)
Sex			
Male	15 (25.0)	5 (16.7)	20 (22.2)
Female	45 (75.0)	25 (83.3)	70 (77.8)
BRAF V600E mutation			
BRAF (+)	24 (40.0)	–	–
BRAF (−)	36 (60.0)	–	–
Tumor size			
< 2 cm	37 (61.7)	–	–
≥2 cm	20 (33.3)	–	–
TNM Staging			
I	46 (76.7)	–	–
II	3 (5.0)	–	–
III	7 (11.7)	–	–
IV	2 (3.3)	–	–
Focality Status			
Unifocal	11 (18.3)	–	–
Multifocality	19 (31.7)	–	–
Extracapsular invasion			
Yes	14 (23.3)	–	–
No	45 (75.0)	–	–
LNM			
Yes	27 (45.0)	–	–
No	32 (53.3)	–	–
Lymphovascular invasion			
Yes	8 (13.3)	–	–
No	51 (85.0)	–	–
Variant			
Classic	50 (55.6)	–	–
Follicular	7 (7.8)	–	–
Hürthle cell	1 (1.1)	–	–
Sclerosing	1 (1.1)	–	–

*TNM* tumor node metastasis, *LNM* lymph node metastasis

### MMP-9 mRNA levels in the study specimens

**Fig. 1:**
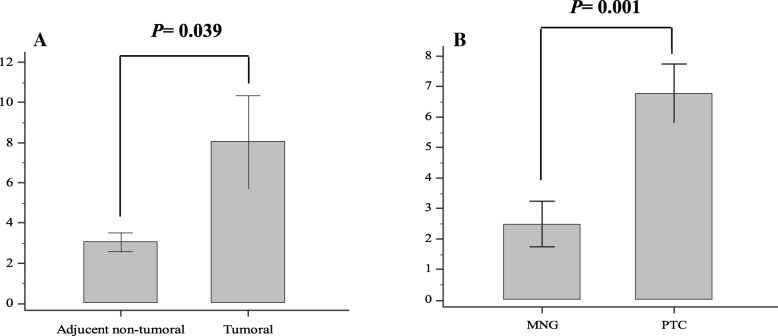
The mRNA level of MMP-9; **a** in tumoral compared to adjacent non-tumoral tissues and **b** in PTC patients compared to MNG, PTC: papillary thyroid carcinoma; MNG: multinodular goiter; error bars were defined as 1 SEM of the mean

The MMP-9 mRNA level was significantly elevated (*P* = 0.039) in tumoral compared to the adjacent non-tumoral tissues; moreover, this higher level was observed in PTC patients compared to MNG participants (*P* = 0.001) (Fig. 1 and Table 3).

**Table 3:** Association of MMP-9 mRNA levels with pathology/clinical characteristics of the study participants

Variables	Mean ± SD	Percentiles			*P value*
		25th	50th (Median)	75th	
Patients					
MNG	2.49 ± 3.70	0.17	0.70	3.40	0.001
PTC	6.77 ± 7.16	1.22	3.83	10.50	
PTC patients analysis					
Tissues of PTC patients					
Adjacent non-tumoral	3.06 ± 3.63	0.32	1.68	4.35	0.039
Tumoral	8.05 ± 16.48	1.07	2.77	6.07	
Age					
< 45 years	1.83 ± 2.13	0.25	0.91	2.86	0.015
≥45 years	6.59 ± 13.01	0.92	2.42	6.08	
Sex					
Male	1.47 ± 1.49	0.40	0.85	2.80	0.993
Female	4.51 ± 10.71	0.23	0.96	2.51	
BRAF V600E mutation					
BRAF (+)	1.66 ± 1.69	0.28	1.25	2.45	0.987
BRAF (−)	4.14 ± 12.59	0.31	0.80	3.64	
Tumor size					
< 2 cm	1.65 ± 1.68	0.35	1.18	2.46	0.960
≥2 cm	5.15 ± 14.82	0.23	0.77	2.81	
TNM Staging					
I/II	1.80 ± 1.98	0.28	0.89	2.82	0.011
III/IV	9.23 ± 15.80	1.32	5.53	7.78	
Focality Status					
Unifocal	1.76 ± 1.44	0.43	1.42	3.48	0.481
Multifocality	2.27 ± 5.08	0.30	0.84	1.93	
Extracapsular invasion					
Yes	10.60 ± 22.88	0.55	1.71	4.34	0.354
No	2.06 ± 2.16	0.28	1.35	3.55	
LNM					
Yes	2.82 ± 6.18	0.35	1.08	3.12	0.413
No	1.28 ± 1.27	0.18	0.70	2.12	
Lymphovascular invasion					
Yes	4.52 ± 0.82	3.65	4.81	5.10	0.003
No	1.65 ± 1.60	0.40	1.08	2.70	

*PTC* papillary thyroid carcinoma, *MNG* multinodular goiter, *TNM* tumor node metastasis, *SD* standard deviation, *LNM* lymph node metastasis, *P value* is for median of MMP-9 expression

### Association of MMP-9 mRNA level with different clinicopathological characteristics of PTC patients

**Fig. 2:**
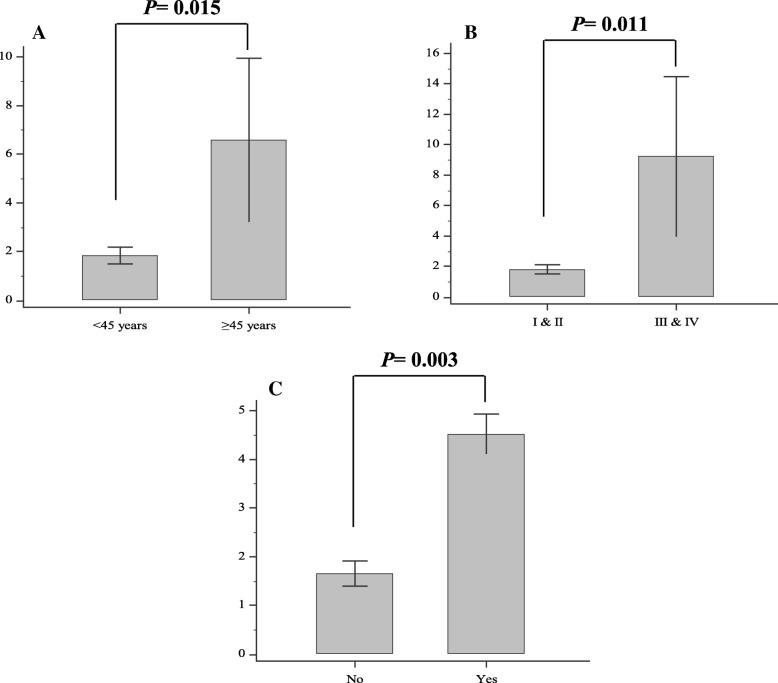
The mRNA level of MMP-9; **a** in PTC patients aged≥45 years compared to age < 45 years, **b** in PTC patients with higher TNM stages (III & IV) compared to lower stages (I & II), and **c** in PTC patients with lymphovascular invasion (yes) compared to those without invasion (no); PTC: papillary thyroid carcinoma; TNM: tumor node metastasis; error bars were defined as 1 SEM of the mean

The mRNA level of MMP-9 was significantly increased in cases aged≥45 years compared to those below this age (*P* = 0.015). In addition, the MMP-9 mRNA level was significantly risen in patients with higher tumor stages (III/IV) compared to those with lower stages (I/II) (*P* = 0.011); also, in patients with lymphovascular invasion compared to those without invasion (*P* = 0.003). There was no significant association of MMP-9 mRNA level with BRAF V600E mutation, sex, tumor size, focality, extracapsular invasion and LNM (only significant results were plotted; Fig. 2 and Table 3).

### Association of MMP-9 protein level with different clinicopathological characteristics of PTC patients

The protein level of MMP-9 was significantly increased in tumoral compared to adjacent non-tumoral tissues (*P* < 0.001); this increase was also seen in PTC patients compared to MNG participants (*P* = 0.004). Moreover, the MMP-9 protein level was significantly higher in patients aged≥45 years (*P* = 0.001), with higher TNM stage (III and IV) (P = 0.001) and with lymphovascular invasion (*P* = 0.036). Other clinicopathological features including BRAF V600E mutation, sex, tumor size, focality, extracapsular invasion and LNM had no significant association with MMP-9 protein level (Table 4).

**Table 4:** Association of MMP-9 protein levels with pathology/clinical characteristics of PTC patients

Variables	Mean ± SD	Percentiles			*P value*
		25th	50th (Median)	75th	
Patients					
MNG	218.14 ± 113.74	119.57	226.51	314.39	0.004
PTC	429.60 ± 288.54	194.88	429.60	467.42	
Tissues of PTC patients					
Adjacent non-tumoral	223.15 ± 137.68	107.27	223.15	268.00	< 0.001
Tumoral	429.60 ± 288.54	194.88	429.60	467.42	
Age					
< 45 years	245.01 ± 258.08	90.34	116.59	259.65	0.001
≥45 years	570.55 ± 186.12	437.51	570.55	624.80	
Sex					
Male	276.52 ± 269.39	71.92	188.94	489.00	0.986
Female	310.36 ± 361.46	89.21	113.30	471.37	
BRAF V600E mutation					
BRAF (−)	256.67 ± 290.37	82.77	100.19	404.35	0.233
BRAF (+)	345.20 ± 380.80	104.74	179.27	505.23	
Tumor size					
< 2 cm	302.53 ± 322.00	77.18	112.10	637.68	0.156
≥2 cm	364.04 ± 354.49	156.08	267.42	399.34	
TNM Staging					
I/II	220.79 ± 220.09	85.35	116.59	257.37	0.001
III/IV	673.65 ± 212.97	521.72	673.65	825.58	
Focality Status					
Unifocal	193.61 ± 168.46	71.15	107.25	343.48	0.595
Multifocality	350.86 ± 422.68	82.34	114.49	637.95	
Extracapsular invasion					
No	406.12 ± 217.46	243.05	406.12	480.45	0.931
Yes	500.05 ± 458.36	140.32	500.05	736.08	
LNM					
No	269.10 ± 259.47	98.07	209.34	283.47	0.169
Yes	351.85 ± 254.60	118.68	298.94	637.68	
Lymphovascular invasion					
No	196.44 ± 195.59	79.04	108.42	227.12	0.036
Yes	453.35 ± 345.74	178.74	298.94	805.16	

*PTC* papillary thyroid carcinoma, *MNG* multinodular goiter, *TNM* tumor node metastasis, *SD* standard deviation, *LNM* lymph node metastasis, *P value* is for median of MMP-9 expression

### Logistic regression analysis results

The association of MMP-9 expression with PTC, TNM stage and lymphovascular invasion (as distinct variables) was assessed by logistic regression analysis adjusted for age (as covariate). The logistic regression outputs are illustrated in Table 5; elevated expression of MMP-9 increased the risk of PTC (OR = 1.20, 95% CI: 1.04–1.37; *P* = 0.009), having higher TNM stage (III/IV) (OR = 1.83, 95% CI: 0.99–3.37; *p* = 0.050) and having lymphovascular invasion (OR = 3.97, 95% CI: 1.68–9.40; *P* = 0.002).

**Table 5:** Logistic regression analysis to assess the association of MMP-9 expression and PTC, TNM stages, and lymphovascular invasion

		B	S.E.	*P value*	Odds ratio	95% Confidence Interval
Patients PTC vs. MNG	MMP-9^a^	0.18	0.07	0.009	1.20	(1.04–1.37)
TNM Stages III/IV vs. I/II	MMP-9^a^	0.61	0.31	0.050	1.83	(0.99–3.37)
Lymphovascular invasion Yes vs. No	MMP-9	1.38	0.44	0.002	3.97	(1.68–9.40)

^a^Adjusted for age and sex

### ROC curve as a test of model prediction power

**Fig. 3:**
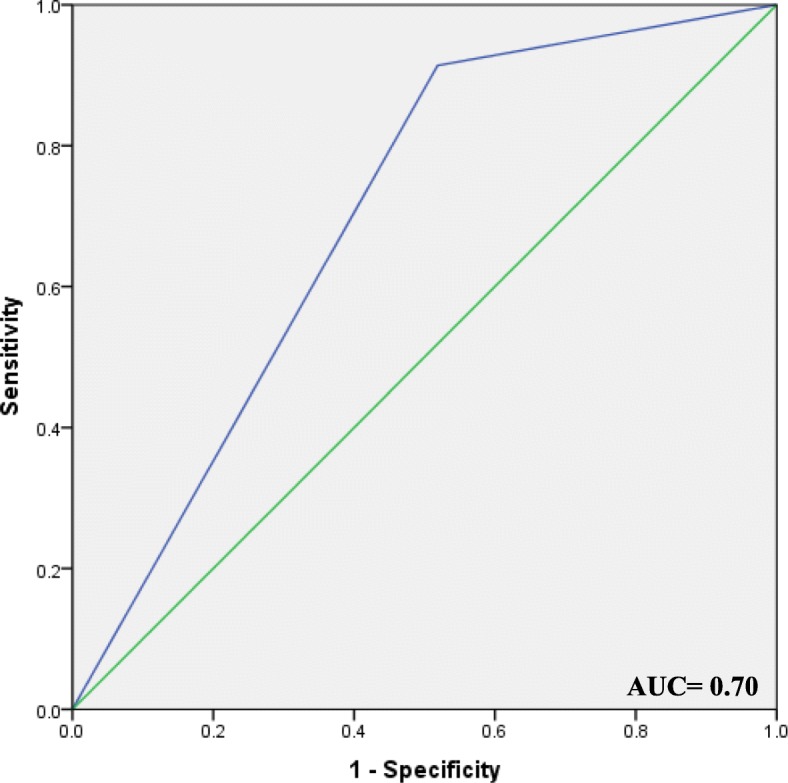
ROC curve analyses of MMP-9 mRNA levels in the discrimination of PTC from MNG patients. The AUC set was 0.70

To evaluate the diagnostic value of MMP-9 mRNA levels for PTC, the ROC curve analysis was performed. The area under the ROC curve (AUC) was 0.70 (95% CI: 0.57–0.83, *P* = 0.003), with 91.4% sensitivity and 51.9% specificity at a predicted probability cutoff value of 0.50 (Fig. 3). PPV and NPV were 85.07 and 66.79%, respectively. This curve and the corresponding AUC show that MMP-9, as a biomarker, has significant prediction power to distinct between PTC and MNG patients.

## Discussion

In the present study, the MMP-9 was significantly overexpressed in tumoral compared to adjacent non-tumoral tissues, and the protein levels showed a significant increase. In addition, the MMP-9 expression in PTC patients was significantly elevated in comparison to MNG participants, also with a significant increase in its protein levels. The MMP-9 mRNA levels in patients aged ≥45 years, with higher TNM stages and lymphovascular invasion was also significantly increased; its protein levels were upregulated as well. No significant association was found between MMP-9 mRNA and protein levels with BRAF V600E mutation and other clinicopathological features. After adjustment for age and gender, logistic regression showed that the odds of MMP-9 expression in the PTC group is about 1.2 times the MNG group; in higher TNM stages, it is 1.83 times the lower stages; and in patients with lymphovascular invasion it is 3.97 times those without invasion.

Although the MMP-9 regulation is not related to transformation of the normal cells into tumoral, it plays an essential role in degrading of type IV collagen (the major component of the basement membrane), thus promoting tumor invasion. Neutrophils, macrophages, or even transformed cells, secrete MMP-9. The extreme secretion of MMP-9 damages tissues in the absence or lack of their inhibitors. In carcinomas, this damage by MMP-9 leads to metastasis and invasion, which are serious challenges.

There are limited studies on the relation between MMP-9 expression and clinicopathological features in PTC. However, in line with our results, Buergy et al. (using the ELISA method) showed that MMP-1 and MMP-9 expressions significantly increase in aggressive FTC or advanced clinical PTC (with extrathyroid invasion, lymph-node metastasis, and high degree of tumor infiltration) compared to adenomas [18].

Although recent studies reported that MMP-9 upregulation is related to BRAF V600E mutation [15], our results showed no significant association between BRAF status and MMP-9 activity in our sample set; this finding is in line with previous reports from other groups in ATC cell lines and colorectal carcinoma [19, 20]. Actually, not all MMP genes have an AP-1 site in their promoter region, regulated by the BRAF-dependent ERK pathway. Other transcription factors, such as STAT, NF-kB and ETS, modulate the regulation of each MMP gene differently [21]. Briefly, BRAF may be involved in the development of tumor via other mechanisms, independent from the MMPs pathway. Furthermore, it seems that the combination of MMP/TIMP at the leading margin of each tumor may be able to create a fine-tuned microenvironment, regardless of MMP expression or baseline oncogenic BRAF mutation. Therefore, further studies should focus on the leading margin of tumors and consider the complete MMP/TIMP system to gain a more detailed insight into the biology of extracellular matrix (ECM) degradation and tumor cell invasion, as a prerequisite for PTC metastasis. Moreover, these contradictory reports may be due to different types of PTC.

In accordance to our results, Huang et al. evaluated mRNA and protein levels of HMGB1, MMP-9 and VEGF-C in 58 PTC, 20 adenomas, 25 MNG and 10 normal participants, using the IHC method; they found that protein levels of MMP-9, VEGF-C, and HUMGB1 were increased in PTC patients. However, in contrast, they demonstrated a positive association with tumor size and LNM, and found no associations with age and gender [22]. Marečko et al. reported a positive correlation between active MMP-9 and LNM, extrathyroidal invasion and degree of tumor infiltration in 120 PTC patients, using IHC and gel zymography. In addition, this correlation was found with the age of the patient, but not with tumor size or gender [23]. Similar to our findings, Kumar et al. assessed MMP-9 activity in some human cancers, including thyroid, breast and colorectal carcinomas; they revealed that this activity was associated with neoplasm growth, invasion and metastasis. Moreover, in thyroid cancer it increased from 33% in stage III to 75% in stage IVA, indicating that MMP-9 was expressed more in advanced stages of malignant diseases [24]. He et al. evaluated MMP-9 and MMP-2 levels in 41 PTC serum samples using ELISA, before and after Radiofrequency Ablation (RFA). They observed that the levels of these two MMPs reduced after RFA; also, they showed that age, degree of calcification, regularity of shape and diameter, and number of foci were effective independent risk factors for the prognosis after RFA [25]. Zhang et al. evaluated the diagnostic values of ultrasounds such as conventional ultrasound (US), contrast enhanced ultrasound (CEUS) and MMP-9 regulation in predicting the cervical LNM in 156 PTC patients, using IHC. They recognized a significant difference in MMP-9 between PTCs with and without cervical LNM; suggesting that combining conventional US, CEUS features and MMP-9 may be useful in predicting the cervical LNM of PTC [26].

Our results indicated that MMP-9 regulation could be used as a potential biomarker to differentiate PTC from MNG, with 70% diagnostic precision; thus, it is suggested that using this gene as a potential diagnostic biomarker for PTC could be more helpful. For further validation of MMP-9’s diagnostic value in the preoperative evaluation of thyroid nodules, analyzing the proposed gene in “intermediate” or “suspicious” FNA cases is required.

Since the pathogenesis of thyroid cancer involves a wide range of molecular disorders occurring throughout life, numerous types of molecular mechanisms in the formation of thyroid tumors have been investigated, including the role of MMPs [27]. MMPs are zinc-ion dependent endopetidases, digesting the underlying membrane and ECM. They take part in a variety of physiological and pathological processes, such as wound healing, angiogenesis, chronic inflammatory disease and tumor growth including metastasis, invasion, and progression [28]. The mechanism by which MMPs contribute to invasion via proteolytic destruction of the ECM (a central event of tumor invasion) is regulating the dynamic interactions between ECM–cell and cell–cell, during migration. Overregulation of MMP-9 has been associated with epithelial to mesenchymal morphological transition [29].

Various efforts have been made to develop new therapies that can control stimulation of MMPs expression in cancerous tissues to block the growth of cancer invasion and metastasis [30, 31]; these findings provide further evidence for MMPs as a suitable target for molecular therapies in aggressive thyroid cancer patients.

Several studies have demonstrated MMPs being involved in the growth and development of thyroid cancer, while there is no agreement on the precise immunolocalization of MMPs, either in stromal cells or tumor cells, or in both. Tumor cell invasion through proteolytic enzymes results in the destruction of cells surrounding the ECM [13, 27, 30]. However, it appears that MMPs are important enzymes involved in local attack and metastatic PTC cancer, being reported in several studies related to different members of the MMP family, including MMP-1, MMP-2, MMP-7, MMP-9, MMP-10, MMP-13, MMP-14 and MMP-15 [13, 32]. The cDNA microarray assessment showed that MMP-2 expression had over two folds increase in PTC tissues compared to benign thyroid tissues. In particular, previous studies have reported an increase in MMP-2 and MMP-9’s mRNA and protein levels in a variety of thyroid cancer cells [30, 33]. Other studies reported that the expression of MMP-2, along with MMP-9, TIMP-1 and TIMP-2, is associated with thyroid tumor invasion and metastasis [13, 34]; these investigations indicate the major role of MMPs expression and the necessity for understanding the mechanism and effectiveness of different factors in thyroid cancer treatment.

Of the current study’s limitations was that we had no information on the follow-up of patients, to evaluate the correlation between MMP-9 expression and persistence of the disease or mortality. We had no access to FNA samples of the studied patients, to perform MMP-9 expression analysis for the diagnosis of PTC.

In conclusion, our findings revealed that the MMP-9 mRNA and protein levels had no association with BRAF V600E mutation in Iranian PTC patients. However, these levels were associated with age, TNM stages and lymphovascular invasion, which were defined as malignant factors. Moreover, elevated levels were observed in PTC patients compared to MNG participants. Therefore, it could be concluded that MMP-9 levels may be a potential biomarker to distinguish PTC from MNG patients.

## Abbreviations

AJCC-TNM: American Joint Committee on Cancer Tumor-Node-Metastasis
AUC: Area under the curve
CEUS: Contrast enhanced ultrasound
CI: Confidence interval
ELISA: Enzyme-linked immunosorbent assay
FTC: Follicular thyroid cancer
IHC: Immunohistochemistry
LNM: Lymph node metastasis
MAPK: Mitogen-activated protein kinase
MMP: Matrix metalloproteinase
MNG: Multinodular goiter
PCR: Polymerase chain reaction
PTC: Papillary thyroid cancer
qRT-PCR: quantitative reverse transcriptase real-time PCR
RFA: Radiofrequency ablation
ROC: Receiver operating characteristic
SD: Standard deviation
